# The Role of Toll-Like Receptors in Retroviral Infection

**DOI:** 10.3390/microorganisms8111787

**Published:** 2020-11-14

**Authors:** Edward P. Browne

**Affiliations:** 1 Department of Medicine, University of North Carolina at Chapel Hill, Chapel Hill, NC 27514, USA; epbrowne@email.unc.edu; 2 Department of Microbiology and Immunology, University of North Carolina at Chapel Hill, Chapel Hill, NC 27514, USA; 3 UNC HIV Cure Center, University of North Carolina at Chapel Hill, Chapel Hill, NC 27514, USA

**Keywords:** human immunodeficiency virus (HIV), Toll-Like Receptors (TLRs), innate immunity, retrovirus, interferon

## Abstract

Toll-like receptors (TLRs) are key pathogen sensing receptors that respond to diverse microbial ligands, and trigger both innate and adaptive immune responses to infection. Since their discovery, a growing body of evidence has pointed to an important role for TLRs in retroviral infection and pathogenesis. These data suggest that multiple TLRs contribute to the anti-retroviral response, and that TLR engagement by retroviruses can have complex and divergent outcomes for infection. Despite this progress, numerous questions remain about the role of TLRs in retroviral infection. In this review, I summarize existing evidence for TLR-retrovirus interactions and the functional roles these receptors play in immunity and pathogenesis, with particular focus on human immunodeficiency virus (HIV).

## 1. Introduction

The innate immune system represents the front line of defense against invading microbes, mediating rapid responses at mucosal surfaces that can prevent the establishment of disseminated infection. This system is comprised of a diverse set of molecules and pathways that serve to sense the presence of invading pathogens, and to inhibit replication of these pathogens. A remarkable feature of the innate immune system is its ability to detect infection by a wide range of pathogens with different characteristics—ranging from viruses to bacteria and fungi, and to initiate rapid expression of anti-microbial genes within hours of infection. These anti-microbial responses can take the form of proteins that target specific steps in viral replication (often referred to as “restriction factors”) or pro-inflammatory signaling pathways and cytokine networks that recruit and activate cell types such as natural killer cells and macrophages that clear pathogens. The signaling pathways activated by the innate immune system in response to infection are complex, and vary depending on the microbe and on the sensing pathway, but typically involve activation of both proinflammatory transcription factors such as NF-κB, and antiviral cytokines such as type 1 interferons (IFNs) [1]. In addition to the induction of rapid anti-microbial gene expression, the innate immune system can also prime and shape adaptive immune responses [2]. In some cases, this impact on cells of the adaptive immune system is direct—B cells and T cells themselves express microbe sensors that regulate their activation, while in other cases, the innate immune system can promote expression of lymphocyte regulating cytokines, or trigger the maturation of antigen presenting cells (APCs), thereby promoting the presentation of antigen to CD4 and CD8 T cells [3]. The vital importance of the innate immune system in the vertebrate defense against viral infection is indicated by the rapid evolutionary rate of many innate restriction factors, and by the profound defects in antiviral responses observed in mice and humans with inactivating mutations in these genes [4]. Extremely high non-synonymous substitution rates for restriction factors over recent evolutionary history, in particular, reflect a relentless and ongoing arms race between pathogens and the host innate immune system [5].

Despite the vital importance of the innate immune system, strict regulation of innate signaling pathways is also essential. Inappropriate or unregulated activation of innate immunity can be highly detrimental to an organism’s health, resulting in chronic inflammatory diseases and autoimmunity [6]. A critical point of regulation for the innate immune system is the initial interaction of the microbe and microbe-sensing receptors. Microbe sensing is mediated by several families of proteins collectively referred to as “pattern recognition receptors” (PRRs). PRRs bind to broadly conserved molecular features of microbes, referred to as “pathogen-associated molecular patterns” (PAMPs), and initiate signaling cascades leading to expression of innate immune response genes [7]. The existence of PRRs that detect broadly conserved characteristics of microbes was originally postulated by Janeway, several years before the discovery of most PRRs [8]. Pattern recognition typically relies on plasma membrane or cytoplasm localized proteins that detect unique classes of molecules found in microbes but not in host cells. For sensing bacteria, for example, products of uniquely bacterial metabolism such as lipopolysaccharide (LPS) and flagellin serve as PAMPs. Detecting viral infection, however, presents a complication to this model, since viruses are assembled inside infected cells, using host cell metabolites. Fundamental discoveries over the last 10–15 years have revealed that the problem of virus sensing has been solved by our immune system in a number of ways – (1) detecting inappropriate localization of nucleic acid, (i.e., DNA in the cytoplasm, or RNA or DNA in the endosome), (2) detecting types of nucleic acid that are unique products of viral replication by an RNA-dependent RNA polymerase—e.g., double stranded RNA (dsRNA) and 5′ triphosphate modified RNA, or (3) detecting perturbations to host cell homeostasis induced by viral replication [9,10].

## 2. Toll-Like Receptors (TLRs)

Over the past 25 years, enormous progress has been made in discovering and characterizing PRRs in the vertebrate immune system. The overall diversity of PRRs is beyond the scope of this review, and has been reviewed elsewhere [11,12]. Briefly, many PRRs fall within three large families of receptors – NOD-like receptors (NLRs), RIG-I-like receptors (RLRs) and Toll-like receptors (TLRs). TLRs represent the best characterized family of PRRs. The prototypical TLR, Toll, was initially characterized in Drosophila, and remarkably, mutation of the Drosophila Toll gene causes enhanced susceptibility to fungal infection—the first indication of an immune role for Toll proteins [13]. Soon thereafter, a human homolog of Toll was discovered and identified as a long sought-after receptor for bacterial lipopolysaccharide (LPS) [14,15]. Other homologs of Toll were soon discovered, leading to the naming of this family as Toll-like receptors. TLRs are type 1 transmembrane proteins and have a conserved structure, consisting of an extracellular domain with leucine rich repeats, and an intracellular domain that mediates signaling. TLRs are found in all vertebrates and many invertebrates, indicating their evolutionary importance. Ten different TLRs are encoded in the human genome (TLR1-10), while 12 TLRs are present in mice [16]. Engagement of microbial PAMPs is mediated by the extracellular domains of the TLRs, and each TLR has evolved distinct binding/sensing abilities. TLR1, TLR2 and TLR6 all recognize bacterial lipoproteins [17], while TLR3 senses double-stranded RNA (dsRNA)—a common intermediate product of RNA virus replication [18]. TLR4 detects LPS [14], while TLR5 is activated by the bacterial protein flagellin [19]. TLR7, TLR8 and TLR9 are nucleic acid sensing TLRs that respond to ssRNA (TLR7/8) and CpG DNA (TLR9) [20,21]. Notably, TLRs diverge in terms of subcellular localization, with TLRs that sense bacterial PAMPs (TLR1, TLR2, TLR4, TLR5, TLR6) localizing to the plasma membrane, while nucleic acid sensing TLRs (TLR3, TLR7, TLR8, TLR9) localize to endosomal compartments. The internal location of nucleic acid sensing receptors likely helps to restrict inappropriate activation by ambient nucleic acid [22]. For a virus to trigger these TLRs, therefore, viral particles must be internalized and trafficked to an endosomal compartment where the viral particles are degraded by host cell enzymes, releasing the viral nucleic acid. Each TLR exhibits distinct patterns of expression, which regulates its overall biological activity [23].

## 3. TLR Signaling

Intracellular signaling by TLRs is initiated by ligand-induced dimerization and/or ligand-induced conformational changes of the Toll-Interleukin-1 receptor homology region (TIR) domains located within the cytoplasmic tail of the TLRs [24,25]. These cytoplasmic domains then mediate assembly, via the adaptors TIRAP or TRAM, of two distinct signaling complexes—the Myddosome or the TRIFFosome [26,27]. These complexes consist of multiple helical copies of either Myd88 and IRAK proteins, or of TRIF. Formation of these complexes then triggers a signaling cascade that results in the activation of a set of transcription factors (TFs) including NF-κB, AP-1 and IRF3, although the precise details of activation for each of these TFs varies depending in the individual TLR, and the cell type in which it is expressed [16]. Although many of the TLRs have been undergoing purifying selection in the genome, indicating a conserved molecular function [28], comparative studies of different mouse strains indicate significant evolutionary plasticity within the TLR signaling pathway [29]. Further details of TLR signaling have been reviewed elsewhere [16,23,30].

## 4. Innate Sensing Receptors for Retroviruses

The retrovirus family has been present in vertebrate species for at least 450 million years [31] and retroviruses are classified into two major sub-families—orthoretrovirinae and spumaretrovirinae. The orthoretrovirinae are further sub-classified into alpharetroviruses, betaretroviruses, gammaretroviruses, deltaretroviruses, epsilonretroviruses and lentiviruses based on sequence similarity. All retroviruses share the basic characteristic feature of a dimeric single stranded RNA (ssRNA) genome that is reverse transcribed into double-stranded DNA (dsDNA) after entry, by a virion-associated reverse transcriptase enzyme. Integration of this reverse transcribed viral DNA into the host cell DNA to form a provirus facilitates stable long-term infection of host cells. Retroviruses represent important human pathogens—the lentivirus Human immunodeficiency virus (HIV) in particular, but also Human T cell leukemia viruses 1 and 2 (HTLV1 and HTLV2) [32]. Foamy viruses, from the spumaretrovirinae family, by contrast, can cause non-pathogenic zoonotic infection of humans [33]. The large number of endogenous retroviral sequences present in the human genome also indicates the historical importance of retroviruses as human pathogens. Numerous retroviral pathogens of non-human animal species have been also identified, including sheep, cows, cats, and horses and even fish and clams [34]. As such, it is unsurprising that retroviruses have played a role in shaping the vertebrate immune system, including through the evolution of retrovirus sensing PRRs. 

The past 15 years have seen dramatic advances in our understanding of how retroviruses interact with host innate sensing pathways. For retroviruses, however, the problem of pathogen sensing is uniquely complicated, due the presence of thousands of retroviral sequences within the human genome. Although human endogenous retroviruses (ERVs) are not actively replicating, many ERVs carry out parts of the retroviral replication cycle and can generate potential intracellular ligands for retroviral PRRs [35]. This creates a conundrum for the human immune system—on one hand the evolution of retrovirus sensing PRRs is essential for protection from exogenous retroviral infection, but these same sensing pathways could predispose the host to ERV-triggered autoimmunity. This problem is illustrated by a rare autoimmune disease, Aicardi–Goutieres syndrome (AGS). AGS is caused by loss-of-function mutations in any one of a number of different genes, and is characterized by a persistent, unrestrained IFNα response in afflicted individuals, leading to severe mental and physical complications [36]. Interestingly, genetic analysis of AGS-related genes indicate that the underlying molecular pathology is triggered by chronic stimulation of a virus sensing pathway that has, in part, evolved to sense reverse transcribed retrovirus DNA [37]. The main role of the AGS genes, it seems, is to dampen the activation of this pathway by endogenous retro-elements, including ERVs. The existence of a set of host genes that can limit sensing of retroviral PAMPs raises the hypothesis that retrovirus sensing pathways have been selected for suboptimal sensitivity, and that this could potentially contribute to ineffective immune responses to exogenous retroviruses such as HIV. The complex relationship between the innate immune system and ERVs is also illustrated by a recent study demonstrating that IFN-sensitive enhancers located with ERVs play a key role in transcriptional regulation of important innate immune response genes [38].

Several genes have been identified as encoding potential PRRs for retroviruses, and the overall diversity of retrovirus PRRs has been reviewed elsewhere [39,40,41]. These studies have revealed a diverse set of retrovirus sensing pathways that respond to a variety of retrovirus-associated molecules–including both viral genomic RNA, reverse transcribed viral DNA, viral proteins, as well as non-viral molecules associated with retroviral particles. This review will focus on in vitro and in vivo evidence for the role of TLRs in the host innate and adaptive immune responses to retroviral infection, with particular focus on HIV.

## 5. Which TLRs Sense Retroviruses?

**Figure 1:**
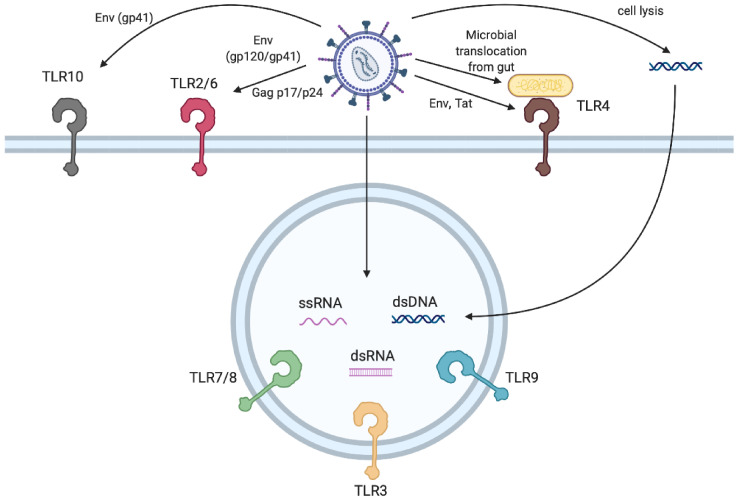
Sensing of retroviral infection by Toll-like receptors (TLRs). Retroviruses can be detected by several TLRs, by both direct and indirect mechanisms. TLR2/6 and TLR10 sense HIV viral structural proteins Gag (p17, p24) and Envelope (gp41, gp120) at the plasma membrane, while TLR4 detects Env or Tat. TLR4 can also be engaged by virion-associated LPS or translocated bacteria from mucosa. Endosomal TLRs are activated by viral ssRNA (TLR7, TLR8), viral dsRNA (TLR3) or by dsDNA (TLR9), possibly of host origin.

**Figure 2:**
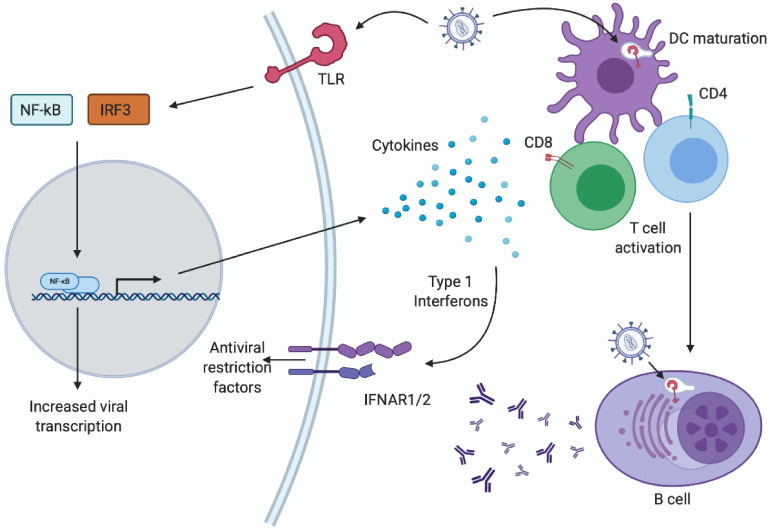
Complex outcomes of retrovirus-TLR interactions. Engagement of TLRs by retroviral particles during infection can have complex and opposing effects on retroviral replication. Activation of a TLR by retroviral PAMPs in an infected cell, either at the plasma membrane, or at an endosomal membrane, triggers activation of NF-κB and IRF3, leading to increased expression of immune modulating cytokines and type 1 interferons (IFNs). NF-κB activation also directly promotes HIV transcription. Cytokines/IFNs can then promote DC maturation and shape T cell activation/polarization. Dendritic cells (DCs) can also directly sense HIV through TLR7 to promote maturation or IFN secretion. IFNs bind to the IFNAR1/2 complex to promote expression of interferon sensitive genes (ISGs) including retroviral restriction factors such as APOBEC3G, BST2 and SAMHD1. B cell intrinsic detection of retroviruses through TLR7 promotes virus-specific antibody responses.

Over the past decade, considerable evidence has accumulated that TLRs play a key role in the immune response to retroviruses. This evidence has come from experiments using a variety of approaches and model systems–biochemical studies of TLR binding, reverse genetic approaches to manipulate TLR expression in cell culture models of infection, and from the analysis of infections in TLR knockout mice. More recently, the analysis of genetic variants in TLRs in humans has provided intriguing clues about the possible roles of TLRs in clinical HIV infection. When considering the evidence for the activity of TLRs in anti-retroviral responses, it is important to distinguish between direct and indirect roles for TLRs in infection. A direct role would consist of cell-intrinsic TLR engagement by viral ligands in a cell type naturally infected by the virus, during the course of an infection cycle. However, cells that are not fully permissive for retroviral infection can still participate in TLR-dependent immunity, and viral entry or internalization is likely sufficient to trigger TLR-dependent immunity, resulting in the release of cytokines or cell-mediated responses that have an indirect or “bystander’ effect on virus infection [42]. Defective virus particles that are not capable of full infection, which greatly outnumber infectious particles, also likely play a major role in TLR engagement during infection [43]. TLRs could also play a role in sensing molecules that are released as secondary consequences of infection—lysis of infected cells releasing free nucleic acids, or microsomes containing TLR ligands, triggering TLR-dependent paracrine effects. Microbial translocation caused by changes in intestinal permeability during acute HIV infection, for example, may trigger TLR4-dependent inflammation and immune activation [44]. In many cases, the relative importance of these direct and indirect pathways remains unclear. To date, evidence has implicated TLR2, TLR3, TLR4, TLR7, TLR8, TLR9, and TLR10 in various models of retroviral infection (Figure 1). The outcomes of these interactions have complex, and potentially opposing, effect on retroviral replication and immunity (Figure 2). In this review, the evidence for the role of each TLR will be considered in turn. 

### 5.1. TLR2

TLR2 forms heterodimeric complexes with TLR1 and TLR6 to detect bacterial lipoproteins [17]. Nevertheless, a number of reports have implicated TLR2 in responses to viral glycoproteins, through an, as yet, unclear mechanism [45,46,47,48,49]. For HIV, both subunits of the envelope glycoprotein (gp41 and gp120) have been shown to bind to and modulate TLR2 activity. Rueven et al. found that the transmembrane (TM) domain of TLR2 interacts with the TM domain of gp41 inside the plasma membrane [46]. Curiously, this interaction inhibited TLR2 signaling, with Env expressing cells exhibiting reduced responsiveness to TLR2 ligands. This observation raises the possibility of TLR2 inhibition as a potential immune evasion strategy for HIV, although the natural TLR2-activating ligand in this model is unclear. By contrast, another study found that gp120 could bind and activate TLR2 in a heparan sulphate dependent manner [45]. Furthermore, gp120, when added to seminal fluid was able to upregulate inflammatory cytokines and promote disruption of tight junctions, and this response could be blocked by TLR2-specific antibodies [45]. These apparently opposing outcomes of the TLR2/Env interaction are curious and have not yet been reconciled. Some evidence also suggests that Gag proteins p17 and p24 can also bind and activate TLR2/1 and TLR2/6 heterodimers respectively [50]. Thus, HIV may encode multiple potential TLR2 ligands. Infection of TLR2 knockout mice with Friend virus (FV), a gammaretrovirus, revealed no impact on viral loads or immunity (E Browne unpublished observation), but genetic evidence from humans has indicated a possible role for TLR2 in HIV infection and disease progression. Specifically, a deletion allele of TLR2 (−196 to −174 Ins/Del) is associated with elevated risk of HIV infection and faster disease progression [51,52]. The mechanism behind how this genetic variant impacts HIV infection is still unclear.

### 5.2. TLR3

TLR3 is a nucleic acid sensing PRR that is triggered by binding to dsRNA [18]. Some virus families, such as reoviruses, possess dsRNA genomes, but dsRNA is also generated as an intermediate during RNA virus replication, when viral RNA-dependent polymerases copy viral genomes. HIV possesses a ssRNA genome and does not encode an RNA-dependent RNA polymerase, but nevertheless contains several highly structured RNA genome regions such as the Trans-activating response (TAR) region and the Rev-response element (RRE). These regions include loops and stems of base-paired RNA that could potentially contain TLR3 ligands. Although TLR3 ligands in the HIV genome have not been clearly identified, some evidence indicates the ability of retroviral genomes to engage TLR3. Notably, a 210 bp region from the Feline leukemia virus (FeLV) long terminal repeat (LTR) was shown to be sufficient to induce TLR3-dependent NF-κB activity in murine fibroblasts [53]. Another study has demonstrated a TLR3-depdendent IP10/CXCL10 response to infection with Xenotropic murine leukemia virus-related virus (XMRV) in two cell lines [54]. Some evidence from Friend virus infection in mice indicates TLR3 may make a minor contribution to the CD8 T cell response to infection [55]. Additionally, some data indicate a possible role for TLR3 in HIV infection of central nervous system (CNS) tissue. Human brain endothelial cells abundantly express TLR3, and HIV induces IL-6 expression in these cells in a TLR3-dependent manner [56]. Interestingly, two recent studies examining highly exposed HIV seronegative individuals have identified a polymorphism (rs3775291) in TLR3 that is associated with resistance to HIV infection [57,58]. This variant encodes a non-synonymous amino acid substitution (Leu412Phe). The mechanism behind the apparent resistance to HIV transmission mediated by this allele is unclear, but expression of this variant is correlated with an increased inflammatory cytokine response following stimulation with a TLR3 agonist, suggesting that an enhanced innate immune response may be involved. A more recent study of HTLV-1 infection indicated no association between this allele and HTLV-1 susceptibility, but did identity a different TLR3 SNP (rs3775296) as having a protective effect [59].

### 5.3. TLR4

TLR4 localizes to the plasma membrane and senses the presence of lipopolysaccharide (LPS), an outer wall component of Gram-negative bacteria. As such, it is unclear if it can act as a direct sensor of retroviral particles. Nevertheless, a handful of studies have indicated that viral components may have intrinsic TLR4 stimulating ability. Two studies demonstrated that the HIV transcription factor Tat could trigger TLR4 and induce IDO-1, TNFα and IL-10 expression in monocytes via an NF-κB dependent pathway [60,61]. HIV Env may also bind and activate TLR4 [45,62]. Genetic evidence from humans also points to a role for TLR4 in retroviral immunity. Specifically, the TLR4 Asp299Gly heterozygous genotype was observed at higher frequency in HIV-1 infected individuals than in healthy controls [63]. Additionally, polymorphisms in TLR4 and CD14, a key signaling partner of TLR4, are both associated with T cell recovery after ART [64].

The majority of natural retroviral infections are initiated at mucosal surfaces, at which commensal bacteria could regulate infection through TLR-dependent pathways. Important evidence to support this hypothesis comes from a study demonstrating that mucosal transmission of mouse mammary tumor virus (MMTV) depended on LPS binding to virions, leading to TLR4-dependent IL-6 and IL-10 secretion [65]. Remarkably, germ-free mice were not susceptible to mucosal MMTV infection, indicating a role for commensal microbiota in retroviral transmission [65]. A major hypothesis regarding HIV pathogenesis also predicts an important role for TLR4. Disease progression for untreated HIV-infected individuals is correlated with generalized immune activation, but the mechanisms driving this activation are unclear [66]. Studies of acute SIV infection in Rhesus macaques, however, suggest that HIV infection promotes a breakdown in the normal barriers that maintain intestinal integrity. During infection, increased permeability of gut tissue leads to translocation of bacterial molecules into the blood stream, including LPS [67]. This increased systemic LPS could potentially drive non-specific immune activation through TLR4. Interestingly, Sooty mangabeys, which are innately resistant to SIV disease, encode a truncated variant of TLR4 that is less responsive to in vitro stimulation [68]. Intestinal pathogenesis during HIV infection may also be mediated by a Vδ2 subset of gut-homing γδ T cells with significantly upregulated Δ42PD1 - a PD1 isoform that can also act as a ligand for TLR4 [69]. Consistent with this hypothesis, HIV-infected individuals have been demonstrated to have elevated levels of these cells, and adoptive transfer of these cells into humanized mice caused inflammatory damage that could be prevented by TLR4 blockade [69].

### 5.4. TLR7

TLR7 binds and responds to ssRNA within endosomal compartments and, given that retroviruses have ssRNA genomes, the role of TLR7 in retroviral infection has received considerable attention. In particular, TLR7 responds to uridine-rich ssRNA sequences [20], and both vRNA and uridine rich segments of vRNA have been examined extensively for TLR7-agonist activity. Early studies examining the innate response to HIV particles in plasmacytoid DCs (pDCs) found that HIV particles trigger IFNα and TNFα secretion by pDCs through a mechanism that required vRNA and endocytosis [70]. In addition to the direct effects on pDCs, the secreted IFNα can also cause bystander activation of other immune cell types [70]. Subsequent studies confirmed the presence of TLR7 ligands in the HIV genome and showed that these ligands could indirectly activate immune cells [71]. Meier et al. showed that these ligands could stimulate cytokine secretion by monocytes and pDCs and cause bystander activation of CD8 T cells in a Myd88-dependent manner [72,73]. Notably, TLR7 is an X-linked gene, and some reports suggest a gender-based difference in responsiveness of pDCs to HIV-encoded TLR ligands. In particular, pDCs from female donors express higher levels of IFNα ex vivo when stimulated with HIV-derived TLR7 ligands, and, interestingly, women exhibit elevated immune activation and more rapid disease progression when corrected for viral load [74]. These observations led to speculation that a TLR7-driven innate immune response may play a key role in immune activation and pathogenesis in HIV infection. Consistent with this hypothesis, chronic overstimulation of TLR7 using dosing with synthetic TLR7 ligands in mice leads to profound immune dysfunction [75]. A key prediction of this hypothesis is that blocking TLR7 engagement during infection would mitigate disease progression or induce viral tolerance. Experiments to test this hypothesis in vivo, however, have produced disappointing results. A study in which SIV-infected Rhesus macaques were administered a dual TLR7/TLR9 blocking agent lead to reduced IFNα secretion by pDCs, but had no effect on viral loads or immune activation, suggesting TLR7/9-independent mechanisms of immune activation [76]. TLR7 activation in cell types other than pDCs may nevertheless be important. Interestingly, one study has found that, although TLR7 is expressed at low levels in T cells, HIV infection activates an anergic gene expression program in T cells that is TLR7-dependent [77]. Furthermore, knockdown of TLR7 in CD4 T cells inhibited HIV replication, indicating that this anergic state promotes virus infection [77]. The outcome of TLR7 engagement during HIV infection may also depend on the subcellular localization of TLR7. One report suggests that early endosome localization leads to an IFNα expression outcome, while lysosomal localization leads to NF-κB activation [78]. Foamy viruses can also be detected by TLR7 in pDCs and Gen2.2 cells [79].

It is important to exercise caution in interpreting studies using isolated TLR ligands from viral genomes, since these molecules may exhibit different properties than intact genomes. Indeed, compared to other RNA viruses such as influenza, the HIV genome has a relatively low abundance of polyuridine tracts, raising the possibility that HIV has been selected for a genome that has a low intrinsic ability to activate TLR7. Consistent with this notion, studies have found that HIV particles are a comparatively poor inducer of cell intrinsic innate immunity [80] (Browne EP, unpublished observations). This modulation of viral genome composition to minimize polyuridine could thus be a previously unappreciated immune evasion strategy for HIV.

Nevertheless, genetic studies from both mice and humans indicate a clear role for TLR7 infection in vivo. Infection of TLR7-deficient mice with the betaretrovirus mouse mammary tumor virus (MMTV) or with the gammaretrovirus Friend virus (FV) have demonstrated that these mice exhibit a profound deficit in their ability to generate anti-retroviral antibodies, highlighting an important connection between innate signaling and the adaptive immune response [42,81]. For MMTV, this response required cell entry and was independent of type 1 IFNs [42]. Conditional knockout of the TLR signaling adaptor Myd88 in different immune cell lineages further highlighted the importance of B cell intrinsic TLR signaling for the anti-retroviral antibody response [81]. The loss of TLR7 also correlated with a dramatic reduction of the germinal center response to infection, indicating that TLR7 activation drives the formation or maintenance of germinal centers during retroviral infection [81]. The role of TLR7 in the antibody response to retroviruses was also highlighted by another study that observed spontaneous reactivation and viremia of an endogenous retrovirus in TLR7-deficient mice [82]. This observation also correlated with a lack of an antibody response to this virus. Interestingly, this study also found that combining the TLR7 knockout with TLR3 and TLR9 knockouts lead to the spontaneous emergence of cancer in the triple-TLR-deficient mice, suggesting a role for TLR3 and 9 in cancer immuno-surveillance. Subsequent work found that TLR7 promotes a rapid wave of IL-10 secretion from CD4 T cells that inhibits early FV replication as well as a non-neutralizing IgM response within days of infection [83]. Notably, during infection of TLR7-deficient mice, plasma cytokines were elevated for most inflammatory cytokines but attenuated for IL-10, indicating the existence of a TLR7-independent pathway promoting inflammatory cytokine secretion. Curiously, CD8 T cell responses are also entirely independent of TLR7 [83]. The identity of the innate sensing pathway that triggers the CD8 T cell response during retroviral infection is still unknown. 

Some evidence from mice also indicates that retroviruses can co-opt TLR signaling to enhance viral replication. Innate-like B1 cells represent a key early target for Friend murine leukemia virus (FrMLV), and mice that are deficient in B1 cells exhibit reduced susceptibility to FrMLV infection [84]. Remarkably, Pi et al. observed that transfer of wild-type B1 cells, but not TLR7-deficient B1 cells, rescued viral infection in B1 cell deficient mice [84]. This phenomenon was found to be due to a B1 cell-intrinsic TLR7-dependent signaling pathway that promotes type 1 IFN synthesis and spread of FrMLV to B2 cells [84]. 

Recently, genetic studies in humans have also pointed to a role for TLR7 in HIV infection. A polymorphism in TLR7 (rs179008) is associated with increased viral loads and altered CD4 T cell counts during infection [85,86] and is possibly associated with lower risk of transmission [87]. Another SNP (rs2074109) may also have a small effect on susceptibility to HIV [88].

### 5.5. TLR8 

Despite mediating an overlapping functional role with TLR7 (ssRNA sensing), TLR8 has received somewhat less attention. Nevertheless, a handful of studies point to an important role for TLR8 in the innate response to HIV infection. Meas et al. showed that endosomal HIV can stimulate cytokine secretion from CD4 T cells in a TLR8-dependent manner [89], while Bernard et al. showed that HIV-derived TLR8 ligands can promote inflammation in macrophages [90]. Similarly, another study has shown that HIV can promote IL-1β expression in a TLR8 and NLRP3-dependent manner in human monocytes [91]. The TLR8 gene in mice is a non-functional pseudogene, but some genetic evidence from humans nevertheless points to a potential role for TLR8 in HIV infection in vivo. Oh et al. analyzed a population of 782 HIV infected and 550 healthy controls for three non-synonymous TLR8 SNPs and identified a SNP in TLR8 (rs3764880) that conferred a protective effect regarding HIV disease progression [92]. Interestingly, in vitro analysis indicated impaired NF-κB activity and modulated cytokine secretions for this genetic variant.

### 5.6. TLR9

TLR9 senses the presence of unmethylated CpG dsDNA and localizes to the endosome [21]. As such, it is not clear that it could act as a direct sensor for retroviruses, which typically only generate viral dsDNA inside the cytoplasm of infected cells during reverse transcription. However, TLR9 could conceivably play an indirect role in detecting infection. dsDNA released by infected cells due to cytopathic effect or virus-induced apoptosis could potentially be internalized by TLR9-expressing cells and activate TLR9 signaling. Altogether, little in vitro evidence exists regarding a role for TLR9 in retroviral infection. Blocking TLR9 activation during SIV infection of macaques has no impact on viral loads or immune activation [76]. Furthermore, TLR9-deficient mice exhibit an apparently normal immune response to Friend virus infection (E Browne unpublished observation). Nevertheless, substantial genetic evidence from humans points to a possible role for TLR9 in influencing the outcome of HIV infection. Specific variants of TLR9 have been shown to influence viral load and also mother-to-child transmission of HIV infection [93,94,95,96,97]. The details of how TLR9 impacts HIV infection, and the nature of the TLR9 ligands responsible, will need to be further elucidated.

### 5.7. TLR10

TLR10 represents an unusual member of the TLR family. Like TLR8, no functional murine homolog exists, so evidence for its role in vivo is limited. TLR10 is not expressed in T cells, but it is expressed on B cells, monocytes, and neutrophils. Also, no natural ligand for TLR10 is yet known. Interestingly, antibody-mediated engagement/activation of TLR10 suppresses pro-inflammatory cytokine secretion, suggesting an anti-inflammatory role [98]. However, this report also indicates that HIV Envelope (gp41) can bind and engage TLR10, leading to IL-8 expression and NF-κB activation [98]. The role of this interaction in infection remains unclear.

## 6. Alterations to TLR Expression and Function during HIV Infection

Untreated HIV infection is characterized by profound perturbations in the host immune system, leading to severe immune deficiency and increased susceptibility to opportunistic infections [99]. Loss of CD4 T cells plays a key role in this pathogenesis, but defects can also be observed in other cell types, including B cells and innate immune cells [100,101]. Given the impact of HIV on the overall immune system, an important question is whether HIV affects expression or functional activity of any TLR members and, if so, whether these effects contribute to HIV immune deficiency. A number of studies have examined alterations to TLR expression during HIV infection, and the overall findings point to increased expression for several TLRs. Heggelund et al. found increased TLR2 expression on monocytes during HIV infection [102]. A subsequent comprehensive analysis of expression levels for all 10 human TLRs in PBMCs during untreated HIV infection observed increased expression for TLR6, TLR7 and TLR8 compared to uninfected controls [103]. Furthermore, when considering only those with advanced disease within the infected group, TLR2, TLR3 and TLR4 also exhibited increased expression during HIV infection [103]. Similarly, Hernandez et al. observed increased TLR2 and TLR4 expression during in vitro infection of mDCs and PBMCs [104]. Additionally, increased TLR2 and TLR4 expression was observed in mDCs from untreated people with HIV (PWH) compared to uninfected donors [104]. Studies of gut biopsies have also found increased expression for several TLRs during viremic HIV infection [105]. The mechanisms behind enhanced TLR expression during HIV infection are unclear. However, one possible explanation for this phenomenon is that it occurs in response to IFNα secretion. IFNα has been shown to promote expression for several TLRs [106], and IFNα levels are elevated in both acute and chronic HIV infection.

The impact of increased TLR expression on HIV infection is unknown. Increased expression may sensitize cells to TLR ligands and boost activation of both innate and adaptive immune cells, leading to improved anti-viral immunity. On the other hand, overactivation of TLRs might contribute to non-productive immune activation, or help create a pool of highly activated CD4 T cells for HIV to infect. Some insights regarding this question have come from non-human primate studies. Analysis of SIV-infected Rhesus macaques found that rapidly progressing infection was associated with elevated TLR expression in CD8 T cells [107]. Furthermore, it was proposed than non-pathogenic SIV infection of old-world monkeys such as Sooty mangabeys is associated with impaired TLR7 and TLR9 signaling [108]. Subsequent studies, however, indicated that infection in old-world monkeys induces an initially potent IFNα response, but that this response is subsequently downregulated and controlled [109,110].

A number of studies have also examined the responsiveness of different immune subsets to exogenous TLR agonists in samples from HIV infected individuals. Many of these studies are difficult to compare, since they use a wide variety of TLR agonists at differing concentrations. Also, it is likely that different results could be obtained, depending on whether the studies examined isolated CD4 cells or heterogeneous cell mixtures such as PBMCs. Results may also differ depending on the stage of infection and on whether the individual is receiving ART or not. Overall, studies examining TLR signaling in the context of untreated HIV infection have indicated mixed results, with some studies reporting increased TLR sensitivity, while others reporting decreased responsiveness. Increased TLR sensitivity could potentially be explained by increased expression of TLRs themselves, or of intermediate signaling proteins during HIV infection. For example, in addition to increased TLR expression, Lester et al. observed increased responsiveness to exogenous TLR ligands in samples from individuals with viremic HIV infection [103]. Similarly, Mureith et al. found that in vitro exposure of monocytes to HIV-derived TLR8 ligands increased the responsiveness of the cells to TLR2 and TLR4 ligands [111]. HIV infection has also been demonstrated to increase the responsiveness of astrocytes to the TLR4 ligand LPS [112]. 

By contrast, studies of whole blood from HIV infected individuals on ART demonstrated reduced IFNα and IL-12 secretion in response to TLR7/8 agonists but that responsiveness was restored during treatment interruption [113,114]. Dhamanage et al. observed that pre-exposure of pDCs to HIV reduced responsiveness to the TLR7 agonist imiquimod [115], while Pereira et al. used a proteomic approach to find that pDCs and cDCs from whole blood from HIV infected donors exhibit delayed responses to several TLR ligands [116]. Similarly, Martinson et al. observed that DCs from HIV infected individuals are less responsive to TLR stimulation than healthy controls [117], and Chang et al. reported that PBMCs from HIV infected individuals had reduced responsiveness to TLR9 agonists compared to healthy donors [118]. The mechanisms of reduced TLR responsiveness in these cells during HIV infection is unclear, although the HIV accessory protein Vpu has been shown to antagonize TLR7/9 signaling in pDCs by promoting the association of the restriction factor BST2 with the inhibitory receptor ILT7, causing its activation [119]. 

## 7. TLR Agonists as Therapeutic Agents for Retroviral Infection

The primary function of the innate immune system is to provide rapid upregulation of genes that inhibit microbial replication. As such, it is of considerable interest whether agonists for TLRs have antiviral activity that might be therapeutically beneficial during retroviral infection. In addition to the rapid expression of antiviral genes such as interferon-stimulated genes (ISGs), activating the innate immune system through TLRs could promote B or T cell responses to HIV, thereby enhancing adaptive immune-mediated clearance. Counterbalancing this hypothesis, retroviruses, including HIV, often exhibit enhanced replication in an inflammatory environment [120], and activated T cells are also key target cells for HIV infection. Furthermore, overstimulation of the innate immune system is a major hypothesis for HIV pathogenesis [44,121]. If this is the case, it is possible that pharmacological stimulation of TLRs in the context of HIV infection might enhance HIV replication or pathogenesis. Nevertheless, numerous studies have examined the impact of TLR agonists on HIV infection in in vitro or ex vivo models, while a handful of reports have examined the in vivo impact of TLR stimulation in the context of SIV infection. Several studies in which TLRs are pharmacologically stimulated in the context of in vitro or ex vivo HIV infection have found that TLR stimulation can inhibit HIV infection, particularly for infection of macrophages or PBMCs. Zhou et al. found that treatment of macrophages with the TLR3 agonist polyI:C inhibits HIV infection [122], while Buitendijk et al. observed that agonists for TLR3, TLR7, TLR8 and TLR9 all inhibited HIV infection and induced IFNα/ISG expression in PBMCs [123]. However, other studies paint a more complicated picture of the impact of TLR agonists on HIV infection—Brichacek et al. found that TLR9 agonists inhibited HIV replication in ex vivo cultures of lymphoid tissue, while TLR5 agonists enhanced infection [124]. The mechanisms by which TLR stimulation can inhibit, or enhance, HIV replication are still largely unclear. Two studies examining ex vivo HIV infection of macrophages have made some progress addressing mechanisms—Wang et al. observed inhibition of HIV infection of primary macrophages by LPS (TLR4), R848 (TLR7) and polyI:C (TLR3) and found that this inhibition was independent of NF-κB and Jak-Stat signaling, but partially required p38 MAPK and JNK [125]. Campbell and Spector observed inhibition of HIV replication in macrophages after TLR8 agonist stimulation, and, interestingly, this effect was dependent on both autophagy and vitamin D signaling [126].

By contrast, studies using more complex experimental systems have highlighted ways in which TLR stimulation could potentially promote viral replication or transmission. Dendritic cells (DCs) can enhance HIV infection of CD4 T cells through capture and internalization of HIV at mucosal surfaces via DC-SIGN, and subsequent formation of a transmission synapse with the T cell–known as “trans-enhancement” of HIV Infection [127,128]. Studies using an ex vivo skin explant model have demonstrated that Langerhans cells (LCs), a specialized skin-resident DC subset, inefficiently promote trans-enhancement as immature LCs, but stimulation with TLR1/TLR2 agonists promotes LC maturation, and significantly increases their ability to trans-enhance HIV infection [129]. Systemic exposure to TLR ligands also leads to elevated immune activation, which may increase HIV replication and contribute to HIV pathogenesis [75,130]. Studies of gammaretrovirus infection in mice have also indicated complex outcomes of TLR agonists dosing on retroviral replication in vivo. For example, a TLR3 agonist, when injected post Friend virus infection, reduced viral loads through an IFNα-dependent manner [131]. By contrast, injection of Friend virus infected mice with a TLR7 agonist (R848) increased viral loads and interfered with virus-specific B cell responses (Browne EP unpublished observation).

## 8. TLR Agonists as Latency Reversing Agents

Despite the success of anti-retroviral therapy in reducing viral loads and restoring lifespan in people infected with HIV, treatment interruption leads to viral rebound within weeks [132,133]. Thus, HIV is able to persist for extended periods of time during therapy, and a key frontier in the HIV field is the elimination of these HIV reservoirs from infected individuals. The mechanisms of HIV persistence on therapy are a subject of active investigation, but one widely accepted mechanism involves HIV entering a state of transcriptional latency in a subset of long-lived memory T cells, allowing the virus to persist for extended periods during ART [134]. These latently infected cells are extremely rare (typically <1 in 10^6^ CD4 T cells), making their direct observation and analysis difficult, yet, sporadic reactivation of these cells reseeds infection, leading to rebound during treatment interruption [135,136]. As such, these cells represent a key obstacle to curing HIV infection and a number of strategies have been proposed for curative elimination of these cells. One such strategy is to reactivate latent proviruses through latency reversing agents (LRAs) followed by elimination of antigen expressing cells by immune clearance methods [137]. The clearance arm of this approach could be achieved by boosting endogenous CD8 T cell immunity, or by dosing with exogenous tools such as virus-specific broadly neutralizing antibodies (bNAbs) [138,139]. Given their ability to activate transcription factor complexes that also promote HIV gene expression (NF-κB, AP-1), TLR agonists have attracted attention as potential latency reversing agents [140]. Furthermore, since TLR stimulation could also potentially enhance virus-specific adaptive immunity, agonizing TLRs might aid both the reactivation and elimination steps of this cure strategy.

Several reports have examined the potential of TLR agonists to serve as both LRAs and immune boosting agents by ex vivo stimulation of samples from patients on ART. Most TLRs are not expressed at physiological levels in CD4 T cells, and as such, the most encouraging results from this approach have come from stimulation of mixed cultures such as PBMCs, suggesting that indirect mechanisms likely play a key role in the LRA activity of TLR agonists. Novis et al. screened a panel of TLR agonists for latency reversing activity and identified Pam3CSK4 (TLR1/TLR2 agonist) as being able to increase HIV transcription and p24 expression in a primary cell latency model [141]. Furthermore, this agent reactivated viral cell-associated RNA in two out of seven samples from ART-suppressed/HIV-infected donors. Analysis of downstream signaling pathways suggested that LRA activity involved NF-κB, NFAT and AP-1. In further studies, agonists for six different TLR complexes (TLR1/2, TLR3, TLR4, TLR5, TLR7, TLR8) were tested for ex vivo LRA activity on PBMCs and isolated CD4 T cells [140]. All agents caused modest but significant increase of viral RNA (vRNA) in PBMCs, while in isolated CD4s, only Pam3CSK4 was active, likely due to CD4 T cell expression of TLR1/2 [140]. A TLR7 agonist (GS9620) has also shown LRA activity in PBMCs from PWH through an IFNα-dependent mechanism [142]. One study has reported that the TLR5 agonist flagellin increased HIV transcription in central memory CD4 T cells infected with an HIV reporter virus [143]. TLR3 ligands have been reported to reverse latency in microglia [144]. Dual agonists that target both TLR2 and TLR7 have also been examined and have shown promising activity [145].

Some recent studies have begun to examine the potential of TLR agonists as curative tools for SIV infection in non-human primates. Notably, Lim et al. found that the TLR7 agonists GS986 and GS9620 were able to induce “blips” of viremia up to 10^3^ copies of vRNA per mL of plasma, after four doses [146]. This study also found a remarkable 75% reduction in SIV DNA in peripheral blood but no difference in time to rebound after treatment interruption [146]. However, other studies have found less encouraging results. Del Prete et al. reported that 12 doses of GS9620 in infected monkeys failed to induce viral blips in plasma and caused no reduction in viral DNA or change to rebound time [147]. A limited number of studies have also examined the impact of TLR agonists following clinical dosing of HIV-infected humans, but these studies have yet to demonstrate clear and consistent impact on either viral gene expression, or on the size of the reservoir. Saxena et al. administered a TLR3 agonist to 12 PWH on ART and observed no change to unspliced vRNA and vDNA in cells [148]. However, Vibholm et al. found that twice weekly subcutaneous dosing with the TLR9 agonist MGN1703 was able to induce modest latency reversal in vivo [149]. However, a follow up study of 24 weeks of dosing in 12 patients found no reduction in reservoir size by vDNA abundance or by quantitative viral outgrowth assay (QVOA) [150]. Overall, it is unlikely that TLR agonist dosing will alone be sufficient to eliminate or reduce the viral reservoir, and combination strategies with other LRAs and/or clearance tools will likely be required. Notably, a recent study in Rhesus macaques that combined the broadly neutralizing antibody (bNAb) PG121 with a TLR7 agonist to treat ART-suppressed SHIV infection was able to inhibit or delay viral rebound after treatment interruption [151]. Although, in this study, ART was initiated very early after infection (7 days) and thus the initial reservoir size was likely very small, this result nevertheless serves as an important proof of concept for reservoir reduction via TLR agonists. Despite these mixed results from in vivo studies, these reports have merely scratched the surface in terms of possible latency reversal and clearance studies with TLR agonists. Different combinations of agonists, dosing regimens, and routes of inoculation need to be fully explored to properly test the potential of TLR agonists as latency reversing tools.

## 9. Summary

Since the discovery of mammalian TLRs 24 years ago, a substantial body of evidence has accumulated that demonstrates that this family of PRRs plays key roles in triggering and shaping the immune response to retroviral infection. These studies have revealed a complex network of interactions and outcomes that can inhibit and, in some cases, enhance infection. TLRs regulate both innate and adaptive immune responses to retroviral infection, and it is likely that both viral and non-viral ligands engage TLRs during infection. Although only a small number of TLRs have been confirmed to directly regulate immunity to retroviruses by analysis of infection in TLR-deficient mice, a combination of biochemical studies, in vitro manipulation, and genetic studies of TLR variants in humans, suggest that a broader set of TLRs contribute to retroviral immunity and pathogenesis. A critical question that remains is whether TLR sensing of HIV during clinical infection is suboptimal for host immunity, either due to the low intrinsic TLR-agonist properties of the virus, or due to viral interference with TLR activity or signaling. If this hypothesis is correct, then this raises the possibility that TLRs could be targeted to further enhance immunity to HIV, and/or that disruption of TLR function by infection contributes to HIV-induced immune dysfunction. Although the limited attempts so far to pursue pharmacological targeting of TLRs in HIV infection have produced mixed results, a more thorough effort to test the potential of TLRs for HIV therapy will be required. A deeper understanding of TLR-retrovirus interactions will likely lead to improved strategies to modulate TLR activity in vivo and improve clearance of HIV. In particular, determining the precise molecular mechanisms by which genetic variants of TLRs in humans impact the outcome of HIV infection or regulate HIV transmission will be of the highest importance. Due to the technical challenges of functional studies for genetic variants in humans, the application of genetic tools to small animal models of HIV infection, such as humanized mice (huMice) may permit a more mechanistic understanding of the role of TLRs in HIV infection. The mechanisms of TLR dysfunction during HIV infection will also need to be elucidated before tools to reverse this effect can be developed. Furthermore, optimal regimens of TLR agonists will need to be identified and fully tested for therapeutic benefit during active and ART-treated HIV infection. Cross-talk and interactions between TLRs and other innate retrovirus sensing pathways will also need to be examined. In summary, although TLRs are now clearly established as key regulators of the anti-retroviral immune response, much remains to be learned about how TLRs impact diverse aspects of retroviral infection and the host immune response.
